# The Italian energy transition in a multilevel system: between reinforcing dynamics and institutional constraints

**DOI:** 10.1007/s41358-021-00306-y

**Published:** 2021-12-23

**Authors:** Maria Rosaria Di Nucci, Andrea Prontera

**Affiliations:** 1 Environmental Policy Research Centre, Freie Universität Berlin, Ihnestr, 22, 14195 Berlin, Germany; 2 Department of Political Science, Communication and International Relations, University of Macerata, Don Minzoni Street, 2, Macerata, Italy

**Keywords:** Italy, Multilevel energy governance, Decentralisation, Energy transition, Renewable energy communities

## Abstract

The article analyses drivers as well as coordination mechanisms and instruments for the energy transition in Italy from a multilevel governance perspective. It addresses the structural constraints that influenced the decision-making processes and organisation of the Italian energy sector and the socio-technical challenges opened up by enhancing renewables. The current energy system is making the move from a centralised, path-dependent institutional and organisational structure to a more fragmented and pluralistic one. Renewables and decentralised patterns of production and consumption are key elements of this paradigmatic shift, which is paralleled by a multiplication of decision-making arenas and actors. These actors follow different interests, problem understandings and green growth narratives, increasing the complexity of governing the energy transition. Against this background, community-based renewable energy policy is assuming a very important role and Italy is putting efforts to establish an enabling framework in line with the requirements of the European Union. The goal of this strategy is to foster a positive link between acceptance of the energy transition and decentralised local activities. In the conclusion we address problems and barriers to new modes of governance, and discuss possible approaches to improved cooperation.

## Abstract (de)

Der Artikel analysiert Treiber sowie Koordinationsmechanismen und Instrumente für die Energiewende in Italien aus einer Multi-Level-Governance-Perspektive. Er befasst sich mit den strukturellen Barrieren, welche die Entscheidungsprozesse und die Organisation des italienischen Energiesektors beeinflusst haben sowie mit den sozio-technischen Herausforderungen, die sich durch den Ausbau der erneuerbaren Energien ergeben. Das derzeitige Energiesystem befindet sich auf dem Weg von einer zentralisierten, pfadabhängigen institutionellen und organisatorischen Struktur zu einer stärker fragmentierten und pluralistischen Struktur. Erneuerbare Energien und dezentrale Produktions- und Konsummuster sind Schlüsselelemente dieses Paradigmenwechsels, der mit einer Vervielfachung der Entscheidungsarenen und Akteure einhergeht. Diese Akteure folgen unterschiedlichen Interessen, Problemverständnissen und grünen „Wachstumsnarrativen“, was die Steuerungskomplexität der Energiewende erhöht. Vor diesem Hintergrund nehmen die Bürgerenergie im allgemeinen und Erneuerbare-Energie-Gemeinschaften im Besonderen eine wichtige Rolle ein. Derzeit unternimmt die Regierung Italiens Anstrengungen, einen Förderrahmen zu schaffen, der den Anforderungen der Europäischen Union entspricht. Das Ziel dieser Strategie ist, eine positive Verbindung zwischen der Akzeptanz der Energiewende und dezentralen lokalen Aktivitäten zu fördern. In der Schlussfolgerung gehen wir auf Probleme und Barrieren für neue Formen der Governance ein und diskutieren mögliche Ansätze für eine verbesserte Kooperation.

## Introduction

The Italian energy transition is widely affected by multi-level dynamics. The impact of these dynamics, however, is ambiguous. As in France, direct state intervention in industrial policy (e.g. through ownership and enhancement of national champions) and centralised models had been considered for long time key to achieve economic development and international competitiveness. Over the years and in the era of the liberalisation of the energy sector, centralised frameworks showed their flaws and are nowadays no longer considered to be able to meet the challenges of the energy transition. The decentralisation of energy policy-(making) started in the late 1990s coupled with generous support schemes have been instrumental in fostering the growth of renewables. Decentralisation has favoured the emergence of bottom-up experimentations as well the activation of mutually reinforcing dynamics that have increased the scope and reach of the Italian energy transition. At the same time, the rearrangement of competences over energy policy-making—especially after the 2001 constitutional reform that has included ‘energy’ as matter of competitive legislation between the state and the Italian regions—has somehow caught up Italian renewable energy policy. The country can be described as a ‘quasi-federal’ state in this policy field (e.g. Breton and Fraschini 2016). Conflicts and legal disputes driven by different strategic and economic interests among regions, local governments and the central government on energy matters provoked delayed decisions. In particular, the length of the procedures for the localisation and authorisation of renewable energy plants represents nowadays an issue that could provoke a standstill of the overall Italian energy transition strategy.

By focusing on renewables in the electricity sector, this article analyses barriers, drivers as well as coordination mechanisms and instruments for the energy transition in Italy from a multilevel governance perspective. In particular, we look at the vertical (inter-institutional) dimension of multilevel governance. We ask whether and to what extent decentralisation has been (partially) beneficial to the sector as the lack of effective coordination and scarce public involvement combined with incoherent views on energy transition are dampening the Italian renewable energy policy. Moreover, we ask whether and how innovative bottom-up approaches involving local actors—i.e. energy communities—can help enhancing energy transition pathways in Italy and foster a positive link between acceptance of (renewable) energy projects and decentralised local activities. Indeed, these bottom-up approaches are regarded as a strong means to implement a more democratic and inclusive energy transition (Becker et al. 2017, Szulecki and Overland 2020).

The article is structured as follows. Sect. 2 sketches the analytical framework which draws on recent studies that combine approaches from multilevel governance with insights from research on federalism to investigate the enhancing or hindering effects of multilevel settings on the energy transition (e.g. Ohlhorst 2015; Balthasar et al. 2020). Sect. 3 provides the historical background, illustrates the evolution of the Italian energy sector, its key actors and the current energy mix. It elucidates the developments of the Italian energy policy by focusing on the recent, continuing passage from a centralised energy system dominated by state-owned companies towards one with a growing role for market dynamics and sub-national actors. Sect. 4 analyses from a multi-level perspective the governance and policy of renewables in Italy. It discusses the implications of the Italian ‘quasi-federal’ system for the energy transition focusing on the support schemes promoting renewables in the electricity sector (RES-E), the efforts of regional and local governments on the matter and the coordination mechanisms in place. This section highlights both those reinforcing dynamics that have contributed to foster the Italian energy transition and those negative policy feedbacks hindering the process. Sect. 5 focuses on the current quest for democratisation of the system and the emergence of community energy and energy communities as a major bottom-up innovation and as an answer for the acceptability at local level of renewable energy pathways. In the concluding section we address problems and barriers to new modes of governance, and discuss approaches to improve cooperation.

## Energy transition in multilevel systems: between reinforcing dynamics and institutional constraints

The multi-level governance approach was developed during the 1990s from research on European and international politics (Piattoni 2009). In this period, a trend towards decentralisation at the domestic level was paralleled by the reinforcement of supranational structures of governance, especially in the European Union (EU). This dual movement implied a reallocation of authority from the central state to other centres of governance. According to the multi-level governance approach, however, this process did not diminish the problem-solving capacity of states although it opened the room for new political dynamics, new patterns of horizontal (inter-institutional) interactions as well as new formal and informal mechanisms for coordination across scales of governance (e.g. Hooghe and Marks 2003; Bache et al. 2016).

Scholarly literature has widely recognised that sustainability policies and energy transition take place in multi-level institutional settings (Ostrom 2010; Svedin et al. 2010; Ohlhorst 2015; Homsy and Warner 2014; Ehnert et al. 2018). Actions at the sub-national level, decentralised and bottom-up initiatives are important drivers for achieving ambitious climate targets. For example, in federal states like Germany, Austria, Belgium (see Wurster and Hagemann 2020), and outside Europe like Canada and the US, the sub-national levels have been instrumental in fostering the diffusion of low-carbon policies (e.g. Murray and Niver 2020). Sub-national and local actors can more easily adapt innovations to the needs of local contexts, circumventing the limits on ‘one-size for all’ solutions (Ehnert et al. 2018). Also, experimentations and bottom-up innovations can emerge from lower level and be scaled-up through learning and emulation (e.g. Fuhr, Hickmann and Kern 2018).

The concept of ‘multi-level reinforcement’ claims that the diffusion of new green technologies can gain momentum if governance and policy objectives at various administrative levels complement one other and are synchronised by activating mutually-reinforcing cycles between policy instruments, targets and commitments (Jänicke 2014; Jänicke and Quitzow 2017). Interactive dynamics in systems of multilevel governance can trigger positive policy feedbacks that increase the support for the policy and its effectiveness. Moreover, ‘multi-level reinforcement’ can compensate for the lack of resources at a certain level of governance as interested political actors can mobilise to anchor their policy preferences and ambitions at another level. For example, research on energy transition and climate policies in the EU has shown that sub-national governments have been able to ‘reinforce’ their local actions drawing from financial resources and/or expertise and administrative capabilities at an upper-level (Jänicke and Quitzow 2017; Domorenok and Prontera 2021).

Overall, the literature on multilevel-governance and research on climate policy tend to highlight the positive and enhancing effect of multi-level institutional and political settings on climate action and the diffusion of low-carbon technologies. However, studies on federalism tend to warn about the challenges posed by decentralisation on policy making and policy implementation also with regard to the energy transition (e.g. Scharpf 1988; Ohlhorst 2015; Balthasar et al. 2020; Karapin 2020). Interactive policy-making can trigger negative policy feedbacks that undermine rather than reinforce policy development. States’ problem-solving capacity can be hindered by conflicts of interests and veto players causing stalemates situations or implementation deficits. Coherence and coordination between the national and the sub-national levels cannot be taken for granted also because political actors can have different perspectives on energy transition policies as well as because of the persistence of different incentives at different levels. Formal and informal mechanisms for vertical intergovernmental coordination are usually established in federal systems to find a balance between the autonomy granted to the sub-national units and the needs of coherence and effectiveness for those polices that involve several actors at different territorial scales.

Italy is not a federal state. However, since the early 1990s, a process and decentralisation of powers has taken place in the country. This process has been reinforced by the 2001 Constitutional reform that has widely enhanced the role of the Regional (and local) governments in several policy areas granting them legislative and administrative functions. As a result, Italy has been depicted as a state with a ‘quasi-federal’ institutional structure (Breton and Fraschini 2016). Italy has followed a ‘zigzag path to federalism’ (Levi 2009, p. 6) that has left unsolved several problems concerning intergovernmental (vertical) coordination and the implementation of policies with a cross-cutting and cross-level character (Baldini and Baldi 2014; Breton and Fraschini 2016). Conflicts between the national and the sub-national levels have increased, including legal disputes in front of the Constitutional Court, causing delays and deadlocks in policy developments. A new and comprehensive Constitutional reform was proposed in 2014 to address some of these issues also including the reform of the Italian upper chamber (the ‘Senato’). According to this proposal, the new upper chamber should consist of sub-national representatives. Indeed, the Regions do not participate in the national legislative process. However, this constitutional reform was rejected by voters through a referendum in 2016.

The Italian institutional and political system has maintained its ‘quasi-federal’ structure also with regard to energy policy making. Energy was among the sectors which were targeted by the decentralisation process started in the 1990s. In this sector conflicts between the national, regional and local governments have increased since that period causing delays and stalemates in policy implementation (Senato 2011). Sarrica et al. (2018, p. 444) tried to find out how views on energy sustainability vary across different scales and detected in their perusal of political debates and newspaper reports both coherence and inconsistencies between discourses taking place at different governance levels. Other researchers, however, have illustrated that the activism of regional governments, bottom-up local initiatives and multi-level reinforcement have been decisive in fostering the Italian energy transition (Brondi et al. 2014; Domorenok and Prontera 2021).

## Overview of the Italian energy sector: historical background, energy mix and key actors

**Fig. 1:**
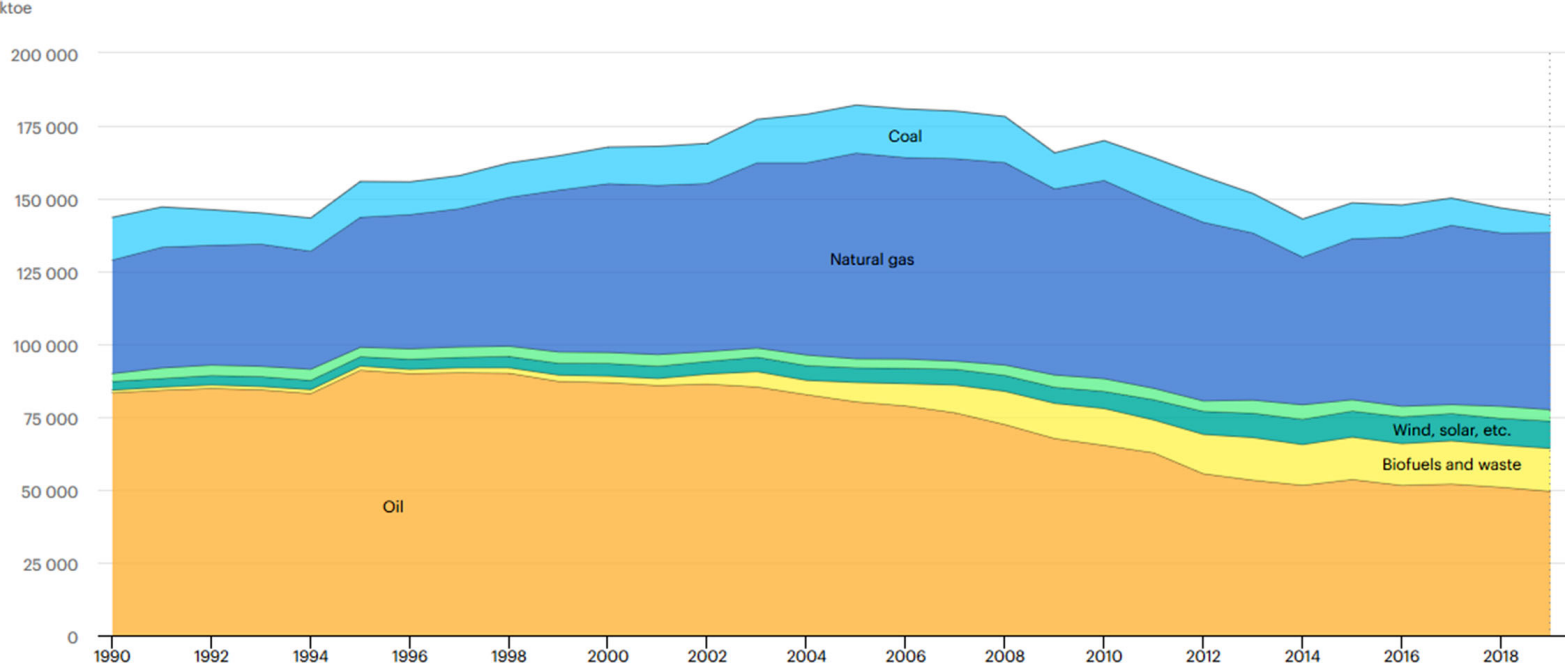
Total energy supply in Italy by source (1990–2019). Source: IEA (2021). All rights reserved

In the last sixty years, the Italian energy system has been characterised by discontinuous and incoherent technology, energy and industrial policy (Di Nucci and Russolillo 2017, 2019). The political parties played for a long time (and to a certain extent still do it) a pivotal role in industrial affairs and policy decisions aimed largely at strengthening the role of one or another industrial group or state-owned enterprises (mostly headed by bureaucrats appointed according to political party proportions) in research and development (R&D) and technology as well as industrial policy (Prontera 2010). Following the nationalisation of the electricity sector in 1962, the establishment of the electricity monopolist ENEL (Ente Nazionale Energia Elettrica) and the reinforcement of national interests in the oil and gas field through prospections and international activities of the growing influential conglomerate ENI (Ente Nazionale Idrocarburi) the two national champions played key roles in setting the course of energy policy till the first wave of liberalisation in the 1990s. Whilst in the late 1950s until the mid-1960s nuclear power was considered as the door to modernity bringing the post war rural Italy a new standing also in research and technology, power games and the interests of the strong national champion ENI directed the development of the energy sector towards hydrocarbons. Due to powerful petroleum lobbies and cheap oil prices, the pursuit of nuclear power was stopped (Di Nucci 2006). The concentration of domestic and external interests developed around the strengthening of ENI also on the international resource market bestowed to Italy to the role of the refinery of Europe. Indeed, fossil energy carriers in particular oil—and later gas—played a key role in the Italian energy mix (Fig. 1). Even after the oil crisis in 1973 when the vulnerability of this system became evident only timid attempts were made to diversify the energy balance. Fossil sources remained paramount for long-run equilibria in the power relations within the energy system (Di Nucci and Russolillo 2019)^1^.

The implementation of planning efforts was hindered by a plethora of regulations, often contradicting each other, by the absence of statutory power to enforce the various plans, by technical weaknesses of the political and ministerial bureaucracy, and by representatives of the political parties serving as presidents or CEOs in state enterprises. This picture has been partly altered by the process of privatisation and liberalisation of the markets in the 1990s as Italy’s energy sector underwent structural adjustments. The reform radically transformed both segments of the market—gas and electricity—which are very different from an economic, institutional and ownership point of view.

**Fig. 2:**
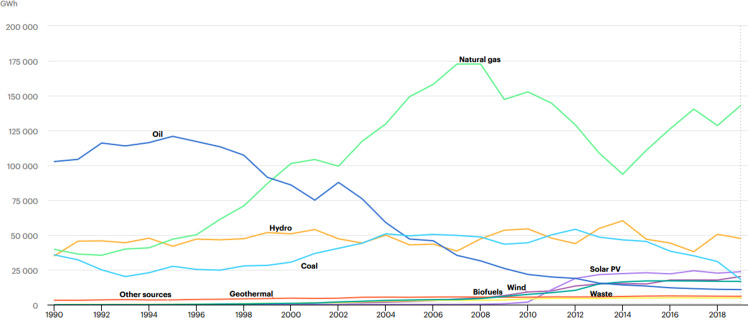
Evolution of the electricity generation by source (1990–2019). Source: IEA (2021). All rights reserved

The so-called Bersani Decree for the reorganisation of the electricity system was passed in 1999 with the aim of liberalising the electricity sector and enhancing competition. In the gas sector, the aim of the so-called Letta Decree, enabling the implementation of EU Directive No 98/30, was to change those features of the sector that no longer met supply requirements in an open market context. In the 2000s, the process of liberalisation and (partial) privatisation of the Italian energy sector continued. These processes were paralleled by the gradual increase of the role of renewables in the Italian energy mix, especially in the electricity sector (Fig. 2). This trend however, has not altered the traditional role played by natural gas, which remains a pillar of the Italian energy system.

Today, energy policy is still dominated by an intertwined system of interactions among the most important institutional actors with a rather complex subdivision of responsibilities. At the institutional level, the key competencies have been rearranged in 2021, when the Draghi Government established a new Ministry for the Ecological Transition, gathering the competences of the former Ministry of the Environment and many of the energy competences of the Ministry of Economic Development. However, a number of responsibilities remain within the Ministry of Economic Development (MSE).

The Environment Ministry coordinates the activities concerning greenhouse gas emissions, the transposition of the EC-directives for emission trading, and is also the co-signer of policy measures promoting renewable energy and energy efficiency within the responsibility of the MSE. The responsibility for biomass lies within the Ministry of Agriculture that promotes the utilisation of RES in agriculture sector and provides financial backing for the cultivation of energy crops. Beside these institutions, some responsibilities also rest within the Ministries of Transport, Finance, Culture and National Heritage. Additionally, CIPE (Interministerial Committee for Economic Planning) which is chaired by the President of the Council of Ministers is responsible for the co-ordination and horizontal integration of national policies. Its competencies, among many others, include climate change. The committee approves national GHG emissions reduction programs. In addition, there is also the Inter-Ministerial Technical Committee for Emissions of GHGs (CTE) established in 2012 to support the CIPE’s climate-related work.

At the sub-national level, key institutional actors are 19 Regions and the two autonomous Provinces (the Provinces of Trento and Bolzano) that are assimilated to regional governments. The role of these actors in Italian energy policy has increased since the 1990s (see below). Coordination between the central level and the subnational level is managed by a ‘System of conferences’ that are institutional bodies designated by the Italian constitution for inter-institutional vertical coordination.

A new influential actor was established following the liberalisation of the energy sector, the regulatory authority for electricity and natural gas (at the time named AEEG). Later its mission (including the setting of tariffs, service quality standards, and the technical and economic conditions for access and interconnections to the networks) was enlarged to include also water and the regulator was renamed in AEEGSI. In the field of research, there are a number of public organisations like the National Research Council (CNR) and the National Agency for New Technologies, Energy and Environment (ENEA) as well as RSE that carries out research in the electricity field with a focus on national strategic projects funded through the Fund for Research into Electrical Systems in energy-efficiency and renewable.

Although ENI and ENEL are still key actors in this sector, new players have entered into the scene as decentralisation has enlarged the spectrum and weight of the relevant actors. New entrants with different stakes in energy decentralisation also increased the complexity of the governance of the electricity sector. Differently from France, where the majority of the French political class remains anchored to the “historical” model based on the role of the State (see Poupeau 2020), in Italy political forces, showed some inclination towards bottom-up approaches. Italian sub-national governments have been proactive in exploiting the opportunities offered by the EU programmes to foster their energy (and climate) agenda (see below). Amongst the new actors we find the transmission system operator and the distribution system operators. Following the unbundling mandated by the liberation, transmission remained a state monopoly and the transmission grid is managed by Terna (and its company Terna Rete Italia) that is responsible for technical rules for planning and operating connections to the grid.

The Bersani Decree also reallocated the management of renewable electricity supply from the ex-monopolist ENEL to GRTN, which later become the Gestore dei Servizi Energetici (GSE). GSE is a publicly owned company that provides incentives for and develops renewables. GSE is also the parent company of Acquirente Unico (AU) that acts as a national wholesaler to protect energy consumers. Finally, Gestore dei Mercati Energetici (GME) is in charge of the electronic platform to exchange power, natural gas and energy tradable certificates (e.g., white certificates for energy efficiency). On the market, the number of distributors and retailers has grown and counts on 128 DSO, eight of which have been unbundled (Eurelectric 2020). However, the concentration is still high with one dominant distributor, ENEL Distribuzione, controlling almost 80% of the network.

**Fig. 3:**
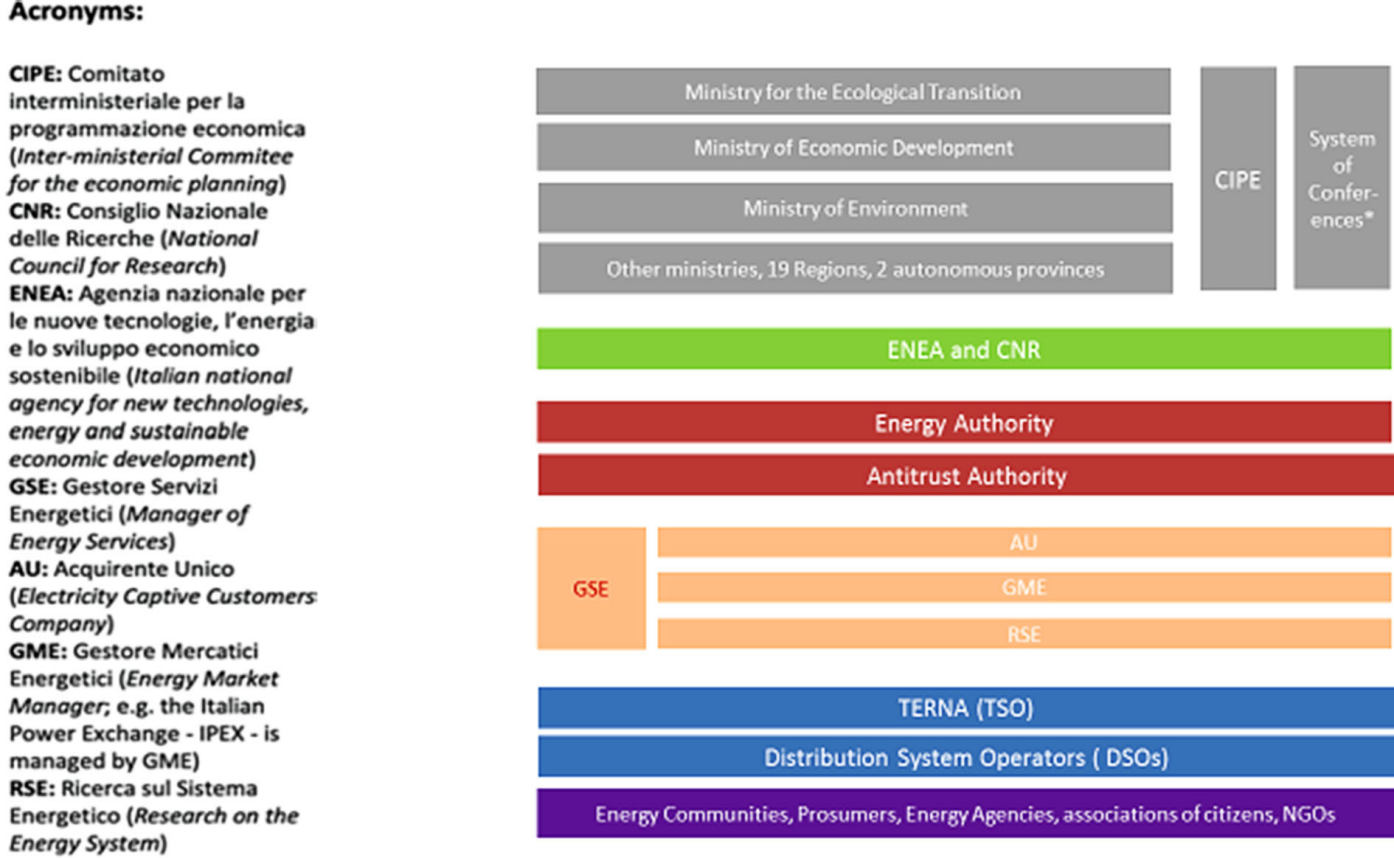
Major actors in the Italian electricity system. Source: adapted from Di Nucci and Russolillo (2019)

Finally, there are over 30 regional and local energy-agencies, established in the framework of the SAVE-Programs of the EC, dealing mostly with energy efficiency, renewables, information, and counselling at local level. These interrelationships and complexity of energy governance in the electricity sector are summarised in Fig. 3.

## Energy transition in a ‘quasi-federal’ state: negative policy feedbacks and mutually reinforcing dynamics

### Energy decentralisation in a ‘quasi-federal’ state

As anticipated, in Italy, the shift from state to market initiated in the 1990s has been paralleled by a shift from the centre to periphery in the area of energy governance. With Law No. 10/1991, the Italian Regions became responsible for formulating Regional Energy Plans (‘Piani Energetici Regionali’). Law No. 10/1991 also provided for energy planning at provincial and municipal levels. Later on, in 2001, with the so-called ‘Turin Protocol’, the Italian regions fully recognised the environmental and climate dimensions of energy policy. Since then, these dimensions have been integrated in Regional Energy and Environmental Plans (‘Piani Energetici ed Ambientali Regionali’, PEAR). In 2001, Italy also enacted an important reform of the Constitution (Constitutional Law 3/2001). This reform modified the Title V of the Italian Constitution granting important functions to Regions over energy matters. In particular, the national level stipulates the general guidelines and principles of the Italian energy policy, and Regions and Provinces legislate accordingly. Within this new institutional setting, the process of administrative decentralisation over the Italian energy policy making has been strengthened and consolidated. Especially in the area of renewables, regional and local governments have obtained a very important role in the authorisation regime for the localisation of RES‑E installations as well as in the environmental impact assessment (EIA) procedures.

The authorisation regime for the localisation of RES‑E installations is regulated by Legislative Decree No. 387/2003 and No. 28/2011. These laws grant Regions and municipalities the competences over all types of RES‑E installations a part from those offshore, which fall under national competencies. Regions are typically responsible for larger installations whereas municipalities for smaller one, although Regions can also delegate functions to the provincial level. The environmental impact assessment over RES‑E installations regulated mainly by Legislative Decree No. 152/2006 and the subdivision of responsibilities follow the same pattern. This fact further contributed to complicate the governance of renewable development. The siting and deployment of renewables plants has become a very long and complex process. Regional and local governments have a veto power over this process, whereas mechanisms for inter-institutional coordination and public involvement have been poorly developed.

Rather than addressing these issues, the national government has sought to recentralise energy policy making. In 2014, the Renzi centre-left government proposed a new Constitutional reform, which among other novelties provided for a recentralisation of decision-making over energy by curtailing the role of the Regions. As the attempts for establishing an upper chamber for regional representatives were spoiled by the result of a failed referendum, the constitutional and institutional setting of the Italian energy policy maintained its quasi-federal structure. This structure is no longer completely centralised because of the redistribution of powers between the State and local authorities, but many features of the old model are still present. The resulting institutional setting has contrasting effects on the Italian energy transition as it triggers both negative and positive policy feedbacks.

### Policy feedbacks and the incoherent development of the Italian RES-E support schemes

**Fig. 4:**
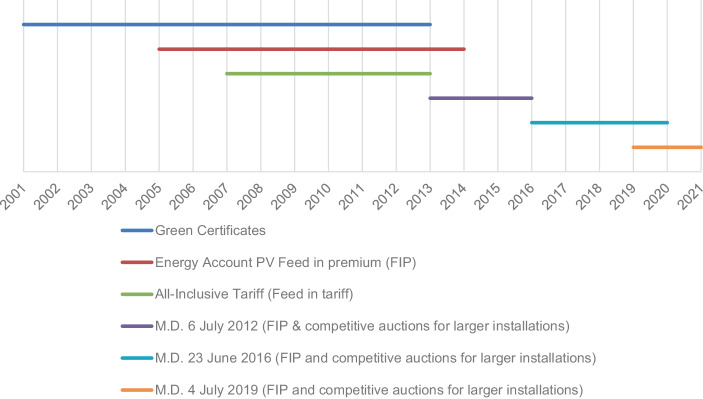
Main Italian Res‑E support schemes (2001–2020). Source: authors’ elaboration from European Commission (2019b). N.B.: *M.D.* Ministerial Decree

Although the evolution of renewable energy in Italy has been characterised by incoherent policies inhibiting the development of a national RES industry, the last decades witnessed a sustained growth of RES. Today Italy has the second-largest solar photovoltaic (PV) installed capacity in Europe, but the achievement of some of the 2020 European targets can be considered to be due to the effects of the economic crisis, rather than to the implementation of coherent policies for the promotion of renewable energy and energy efficiency (Deloitte 2015; Galgóczi 2015, Di Nucci and Russolillo 2019). Indeed, due to the consequence of the COVID pandemic on the Italian economy, in 2020 electricity demand slumped with a 5.3% drop compared to 2019. Renewable energies, which account for around 38% of demand registered a slow development (Terna 2021). The steady growth in the past is due to the generous incentive policy. Starting from the early 2000s, the Italian government has enacted several financial support schemes for RES‑E (Fig. 4)^2^. These policy instruments must be considered within the context of the wider EU obligations and frameworks on energy and climate in which Italian energy governance is embedded. Their implementation, however, must be considered also within the context of the multilevel system of Italian energy governance that emerged from the reforms initiated in the 1990s.

In particular, the development of the Italian support schemes has been affected by multilevel dynamics in two ways. In a first period (2001–2010) the problems related to the authorisation procedures have led the government to grant substantial financial supports for renewables. This strategy was intended to ‘compensate’ developers for the administrative costs and risks they faced in investing in a new sector owing an uncertain regulatory framework. In a second period (2011–2019), however, the very high costs reached by the Italian support schemes prompted the government to introduce stringent measures that resulted in a slowdown of renewables development (Prontera 2021). These two periods and the relative dynamics are further illustrated and discussed by focusing of the development of the main Italian RES‑E support schemes in Fig. 4.

**Fig. 5:**
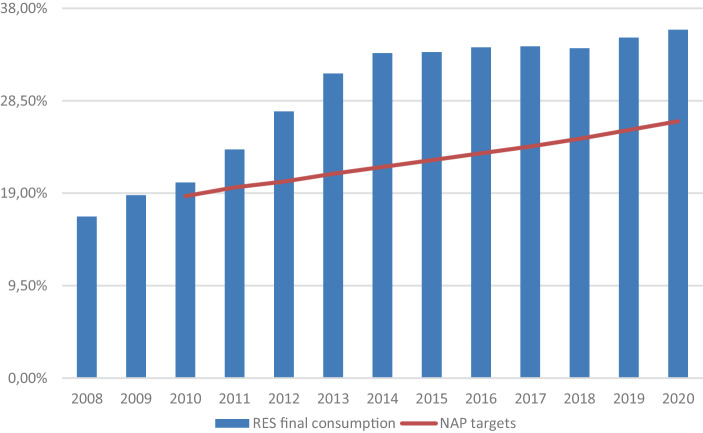
RES as percentage of final electricity consumption and Italian NAP targets. *Source*: authors’ elaboration from GSE (2021)

**Fig. 6:**
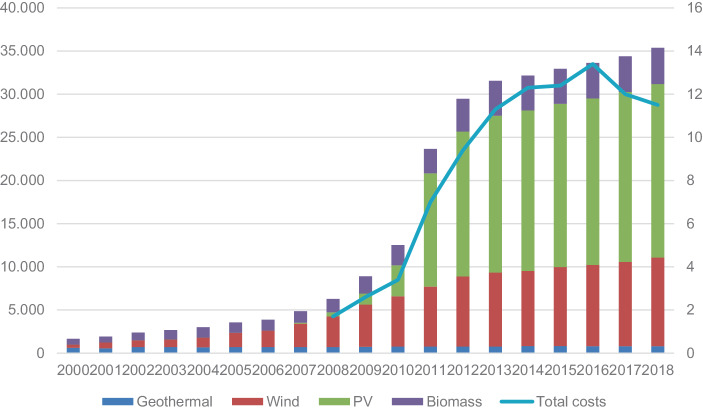
Renewable energy capacity (excluding hydro) installed in Italy (MW, left axis) and total costs for the RES‑E support schemes (in billion Euro, right axis). *Source*: for renewable energy capacity: Terna (2020). For the total costs: ARERA (2020)

The early 2000s witnessed a low level of expansion of renewables despite the introduction of the Green Certificates support scheme which proved unfavourable particularly for PV and smaller installations. In 2005, the Berlusconi government introduced a new feed in premium scheme for PV: the so-called Energy Account (‘Conto Energia’). The subsequent new Prodi government further enhanced the Italian renewable energy policy. Within the context of the implementation of Directive 2009/28/EC, the Italian government set its targets for renewables development in its National Action Plan (NAP): 17% share of renewables in gross final energy consumption by 2020 and 26% for renewables consumption in the electricity sector. To uphold these targets, the government updated the Energy Account, maintaining its generous incentives, and introduced a new feed-in tariff—the so-called All-Inclusive Tariff (AIT) (‘Tariffa Onnicomprensiva’)—for non-PV small generating plants. Also, in 2008, the government introduced some adjustments in order to improve the Green Certificates system and increase the attractiveness of investments in renewable technologies. These innovations proved to be effective in promoting renewables in the country: already in 2010–11 Italy has been able to outdo its EU obligations (Fig. 5). This can be at least in part be ascribed to the very generous incentives, especially for PV. As anticipated, the incentives were set at this high level in order to compensate beneficiaries for administrative burdens and the delays in the authorisation procedures. This situation, however, resulted in a paradoxical development. On the one hand, these support schemes triggered a dramatic increase in renewable capacity between 2008 and 2012, which was exceptional for PV (Fig. 6). On the other hand, this uncontrolled boost produced a parallel explosion of the financial costs of the Italian RES‑E support schemes (Fig. 6)^3^.

The problem of the financial costs of RES‑E support schemes was further aggravated by the negative effect of the 2009 financial crisis on the Italian economy. In particular, the Monti government, with the Ministerial Decree of 6 July 2012, terminated the Green Certificates system and the AIT and replaced them with a new system based on a combination of feed-in tariffs for small and medium-size installations and competitive auctions for larger plants. Additionally, a stringent cap for PV development under the Energy Account was set in 2013. Later on, in 2014, the Renzi government with Decree Law No 91/2014—known as ‘Spalma incentivi’ (spreading incentives)—retroactively reduced the existing feed-in incentives for PV. Overall, the changes made since the early 2010s have been effective in putting under control the costs of RES‑E support schemes, but affected the development of renewables (Fig. 6).

In 2017, the Italian government launched a new National Energy Strategy that set an ambitious target share of RES‑E by 2030 (55%), with a + 80% increase with respect to 2017. The highest contribution was expected from PV and wind power. In 2018, more than 60% of RES energy was supported by public spending (GSE 2021). The reduction of incentives over time (from an average incentive of 290 €/MWh of PV Energy Account to about 50 €/MWh as of o 2016 Decree) dampened the renewables development, which has stalled since the late 2010s (GSE 2020).

In 2019, the Italian National Energy and Climate Plan (NECP) confirmed the 55% RES‑E target for 2030. In the same year, with Ministerial Decree of 4 July 2019, the government further updated the current support schemes (Fig. 4). On the whole, the implementation of the new measures has proven to be problematic, especially for larger installations. Several auctions for new wind and solar plants stayed deserted, and GSE was confronted with the inability to allocate all the planned renewable capacity. Again, the main obstacle has been represented by the complexity and length of the authorisation procedures^4^.

### Mutually reinforcing dynamics and renewables at the regional and local levels

As anticipated, regional and local governments have increased their role in Italian energy policy making since the 1990s. Italian regions have acquired important functions in the following areas: planning; management of programmes for promoting renewables (based on national, region and especially EU funds): policy advice and administrative support for local governments and business actors; licensing and Environmental Impact Assessment (EIA). Local governments are involved in planning, authorisation regime as well as EIA. These latter areas are those that have been more problematic for the Italian energy transition.

With regard to planning, since the ‘Turin Protocol’ of 2001, the Italian regions have included European and national climate and decarbonisation targets and objectives in their planning process and the implementation of related policy measures. This has favoured mutually reinforcing dynamics among EU, national and regional level governance. From 2000 to 2009 all the Italian Regions have issued their PEAR and set targets for renewables development. Regional energy planning has been institutionalised later on as regions started a regular process of revision or updated their plans following the EU and national developments.

This trend has been reinforced with Law No. 13/2009 that has provided for the redistribution of the EU targets on renewables among the Italian regions. The Ministerial Decree of MISE of 15 March 2012 (the so-called ‘Burden Sharing’ Decree) specified the contribution expected by each Region for achieving the national targets. Against this background, all Italian Regions have enacted regional laws and programmes to further support the development of renewables. Moreover, regional governments increasingly involved in their energy planning local stakeholder and business actors. This has facilitated the embedding of energy transition in local development strategies and aided public acceptance for decisions over energy policy.

Regarding the support for renewables, starting from 2007, the EU structural funds begun to play an increasingly important role for implementing regional energy policy. This role has been further enhanced with the 2014–2020 EU cycle of structural funds. The Italian regions have actively used these funds to promote local development strategies based on green economy and renewables. In this case as well, mutually reinforcing dynamics proved to be important in accelerating renewables development. In 2017, almost all the Italian Regions (and the autonomous Provinces of Trento and Bolzano) surpassed the targets assigned them by the national government, the only exceptions being Liguria and Latium.

Regional and Provincial governments have also played an important role providing policy advice and administrative support for local governments and business actors. For example, they have acted as territorial coordinators for the implementation of the EU Covenant of the Mayors and the European Local Energy Assistance (ELENA) programmes in Italy (Domorenok and Prontera 2021). Regional governments also provided policy advice and administrative support to municipal energy planning. Lack of expertise and resources at the local level have long prevented the full implementation of the provision of Law No. 10/1991. However, afterward, the institutionalisation of the regional planning process has facilitated local planning as municipalities have begun to implement measures taken at the regional level as well as defining their planning along with the framework set at the regional level. Moreover, the Covenant of the Mayors has involved a high number of Italian municipalities and business actors, promoting energy and climate policy and local development strategies based on renewables and energy efficiency measures.

### Coordination mechanisms and their limits

Overall, regional and local governments have supported the diffusion of renewables providing financial and other resources. However, with regard to the RES‑E authorisation regime and EIA, the decentralisation of energy policy making has created tensions among the national, regional and local levels. In order to reduce these tensions, increase coherence among the different regional and local procedures and to speed-up the authorisation procedures, the Italian government issued national guidelines in 2011. These guidelines did not change the allocation of decision-making power over renewable energy policy embedded in the constitutional system, although they sought to set a general framework for the regional and local procedures. In 2014, through the so-called unblock Decree (Law 164/2014), the national government sought to re-centralise some legislative and administrative competences in the energy field, including the area of renewables.

These innovations, however, were hardly effective in accelerating authorisation and siting procedures that remain a hurdle for the Italian energy transition. Often, local actors and citizens have a negative attitude towards larger renewables installations. Wind parks or large PV installations—can be at odds with local development strategies as well as with frames of energy transition based on bottom-up practices and decentralised system of production and consumption. Local politics further exploit these tensions which are nurtured by a long legacy of scarce public involvement in energy policy making. These problems are aggravated by an inefficient system of vertical coordination. In particular, the coordination between national and regional levels is assured by the works of the ‘Conferenza Stato-Regioni’, whereas all the administrative actors involved in Italian energy policy (national, regional and local) are involved within the ‘Conferenza-Unificata’. As matter of fact, the general mandate of these bodies proved to be poorly suitable for the challenges posed by the energy transition. They neither provide incentives to reconcile different frames and interests between the national and sub-national actors nor they offer appropriate expertise to support complex technical decisions.

In 2017, the National Energy Strategy provided for the establishment of a special body—gathering different Ministries as well as regional and local governments—with the aim of steering the implementation of the energy transition. This proposal was relaunched with the 2019 NECP, which bolstered a new governance system—with a political body and a technical body—for increasing coordination among national ministries and regional and local governments. These provisions, however, have not been implemented yet and new mechanisms for public consultation are still missing.

## Decentralisation pathways: energy communities and citizens’ energy

As we have described above, the current energy system is in the process of passing from a centralised, path-dependent institutional and organisational structure to a more pluralistic one. The increased share of renewables and decentralised patterns of production and consumption are key elements of the paradigmatic shift, which is paralleled by a multiplication of decision-making arenas and actors. Against this background, community-based renewable energy unfolding different levels of citizens’ participation and ownership of renewable projects has left the niche status and is assuming a more important role. Renewable energy projects led and financed by citizen can be also considered an innovative medium to bridge the investment gap (Pons-Seres de Brauwer and Cohen 2020). At time, Italy is putting efforts to establish an enabling framework for community energy in line with the EU requirements set in the Clean Energy Package. The goal of this strategy is also to foster a positive link between acceptance of the energy transition and decentralised local energy activities.

Renewable and decentralised energy paths emerge as key solutions to achieve the Italian climate and energy objectives and provide a pathway to decarbonisation and increased efficiency of the system (Friends of the Earth et al. 2018; WEC‑I 2020; Legambiente 2021). Local energy communities show high potential for the effective use of distributed energy technologies, including volatile energy production from renewable sources. However, until recently there have been only limited drivers and incentives to boost local energy communities because of numerous limiting factors (Hewitt et al. 2019; Candelise and Ruggieri 2020; Krug and Di Nucci 2020; Standal et al. 2021). As already illustrated, challenges are met already in the planning stage due to complex planning and licensing procedures.

The establishment of community energy initiatives is on the march. Energy communities are seen by some observers as an evolution of former cooperatives which are still the most common legal entities. Italy has a tradition in energy cooperatives, especially in the Alpine areas. In the country, 23 out of a total of 31 electricity cooperatives are located in South Tyrol (Bertolini and Blasi 2021)^5^. In Italy, amongst the domestic drivers for community and citizens’ led initiatives we find on the technological side the strong reduction in the price of the PV technology and on the institutional side the decentralisation and the role attributed to the regional and local governments. Both have played an important role in enhancing the deployment of renewable energy communities. However, significant impulse came in particular from the EU.

The EU Clean Energy Package, including a recast of the Directive on the Promotion of RES (European Commission 2019a) addresses among other things renewable energy communities (REC). In its package ‘Clean Energy for All Europeans’, the EU put the citizens at the centre of the energy transition. The new Internal Electricity Market Directive (2019/944) (IEMD) and the revised Renewable Energy Directive (2018/2001) (RED II) introduced two types of energy communities: Citizen Energy Communities (CECs) and Renewable Energy Communities (RECs) respectively. Both directives grant certain rights and obligations to these communities and provide guidance to member states aimed at promoting such communities^6^. Member States were called to transpose the IEMD by December 2020 and the RED II Directive into national law by the end of June 2021. Italy marked good progress.

It has been claimed that since Italy did not have a specific regulatory framework for collective actions for Renewable Energy Communities (RECs) and off-grid forms of energy production and self-consumption, it was convenient for the Italian government to implement the transposition of the Directive (EU) 2018/2001 (RED II) as soon as possible (WEC‑I 2020). A specific article (Article 42 bis that regulates the establishment of energy communities’ initiatives) was included in the so-called “Omnibus Decree” Law 162/19 (the so-called “Milleproroghe”—thousand postponements) subsequently modified in Law 28/02/2020 n. 8. Legislative Decree 199/2021 implementing REDII was published in the Official Gazette on 30 November and is going to enter into force on 15 December 2021.

For Legambiente, the largest Italian environmentalist association, the implementation of RECs needs to be accompanied by policies to enhance larger renewable energy plants of varying sizes. These not only contribute to improve the Italian energy balance and enable to achieve the goal of zero net emissions, but also, associated with storage systems, will ensure grid flexibility and security (Legambiente 2021). The challenge is to achieve at least 80–100 TWh of production from renewable sources by 2030, while at the same time reducing consumption through efficiency and abandoning fossil fuels by 2040.

Besides national regulations established to implement the legal framework for energy communities, the financial and fiscal support framework has also been refined. The Recovery Decree of 9 May 2020 n. 34, raises tax deductions (Superbonus) up to 110% over five years in the field of energy efficiency and new RES installations. However, this has proved to be detrimental rather than incentivising activities, because of additional bureaucratic procedures and the measures aimed at decarbonisation do not seem to be integral part of a broader, long-term strategy^7^. In September 2020 the MISE issued the decree which defines incentives for collective self-consumption and energy communities and opened up new opportunities. Due to the progress made in the transposition of the RED II Directive, it is now possible to share the electricity produced by RES and electricity consumers will be able to join together.

Whilst policy targets for energy communities are established at national level, regional administrations provide rules and guidelines to implement RES and energy communities in their territories. In this context, the Piedmont Region played a pioneer role and issued the Regional Law n. 12 of 3 August 2018, promoting the institution of energy communities. Following the establishment of the national regulation in 2020, other Regions issued their regulatory framework for energy communities covering some specific local issues while remaining within the context established by national legislation. Regional legislative initiatives have been pursued in Piedmont, Apulia, Liguria, Calabria and Campania.

There are currently various models of community energy ranging from collective self-consumption groups, such as a block of buildings that share a PV system to renewable energy communities as a free association of consumers and prosumers at the level of the same electrical substation. However, there are still only a few examples of energy communities; Legambiente (2021) surveyed around twenty of them.

Although the minimum requirements for an enabling framework (i.e. points a) to e) in RED II Article 22(4)) have been met, there are still issues to be improved. They concern in particular regulatory and capacity-building support provided to public authorities in enabling and setting up renewable energy communities, and in helping authorities to get engaged directly as well as rules to secure the non-discriminatory treatment of consumers that participate in RE (Standal et al. 2021). As Patrucco (2021) remarks, many initiatives aimed at creating wide-ranging Energy Communities, both in terms of territorial extension and socio-technical and economic models are struggling even to launch a small Renewable Energy Community. There are still many uncertainties especially in connection with technical aspects and the difficult relation with distribution system operators. Against this background, it is difficult to find investors for projects of limited size and governed by uncertain regulatory frameworks. Yet, the potential is high and this represents a big driver. According to Legambiente (2021) energy communities could contribute with about 17 GW of new RES power by 2030, corresponding to about 30% of the climate objective to 2030 of the National Energy and Climate Plan (PNIEC). It remains to be seen how the still existing barriers can be removed and they concern specially possibilities of aggregating multiple RECs.

## Conclusions

Starting with the institutional and market changes occurred since the 1990s, the Italian energy policy has moved towards a ‘quasi-federal’ multilevel institutional setting with a growing involvement of regional and local governments. This change has been paralleled by increasing problems, but also opportunities as far as renewables are concerned. The institutional fragmentation regarding the authorisation procedures for siting renewable plants slowed down the RES growth pace. Additionally, the reallocation of several functions to the regional and local levels, along with the instability of the national regulatory framework for the support schemes for renewables have posed several challenges. The administrative burdens have been heavy and, in some cases, ended up hindering renewables development despite the substantial financial incentives provided by the RES support schemes.

Recently, the Italian government has concentrated on creating an infrastructure for supporting sustainable clean generation, with the aim of meeting carbon neutrality by 2050. However, the Italian energy system has been and still is partially **s**ubject to an enduring path dependency, especially because of lock-ins of the following nature: technology (hydrocarbons also used in power plants for electricity generation); infrastructural (large projects); institutional (centralisation) and instrumental (subsidies) (Di Nucci and Russolillo 2019). These lock-ins have proved to be strong barriers to affect changes in the energy system towards more sustainable paths and had serious implications for sustainable and timely policy responses. A path dependence remains especially because of the reliance on gas and there are incongruences and trade-offs in pursuing the gas trajectory and at the same time expanding the integration of renewables in the generation mix. Moreover, the decision for gas also risks to neutralise the aims of the National Energy and Climate Plan in fostering lowest cost clean energy. The plans to build approximately 14 GW of combined cycle turbines to compensate for the loss from the decommissioning thermo-electrical fossil plants has been criticised as inappropriate (see Carbon Tracker 2021) especially since clean energy plants represent a tangible alternative for meeting grid stability and security of supply criteria. Thus, the Italian energy capacity market remains biased in direction of gas.

Against the background of persisting path dependences, the emergence and development of community energy can be seen as a decisive step to enhance the energy transition towards renewables. At national governmental level there are some challenges especially concerning the enhancement of a truly “enabling” framework and the lacking availability of the necessary infrastructure, which puts a stop to many activities or innovative schemes that energy communities are working to implement. But this is not a task for the central government alone. Local governments are also called to enhance measures to promote energy communities in achieving energy efficiency and meeting at the same time also mitigation of energy poverty objectives. Local governments and system operators need to get more acquainted with the specific needs and capabilities of the energy communities. Despite these problems, the process of decentralisation has triggered an activism of regional and local governments. This engagement has been instrumental in promoting positive practices in terms of local energy strategies for enhancing renewables.

In our excursus we have seen that the new Italian multilevel institutional system has been conductive to both negative policy feedbacks that hindered the energy transition, and mutually reinforcing dynamics that fostered the renewables development. The real conundrum concerns the coordination among different scales as coordinated actions are a prerequisite for low-carbon paths (see also Sarrica et al. 2018). Existing mechanisms for vertical coordination have proved to be barely effective. By contrast, collective self-consumption and renewable energy communities can take a prominent place in accelerating the energy transition process by supporting bottom-up practices and increasing the social acceptability of new installations. In this context, the National Recovery and Resilience Plan issued in 2021 as an answer to the COVID-19 pandemic can play an important role to accelerate the Italian energy transition. This plan guarantees the resources necessary to install approximately 2000 MW of new electricity generation capacity in a setting of renewable energy communities and self-consumers of renewable energy acting jointly. But, what is more, the Recovery Plan—as it is described in its name—is also designed to be a resilience plan. This means that, at least on the paper, there are hopes that it can provide new stimuli for the awaited comprehensive reform of the planning and authorisation procedures for renewable plants and thus can represent a decisive step to overcome long-standing barriers.

## Footnotes

1 The existence of this path dependence is proved by the fact that in 1975, as the price of crude oil had already more than quadrupled, the largest order in Europe for oil-fired power plants was placed in Italy (Di Nucci 2006).

2 The first Italian support scheme that covered renewables was introduced already in 1992 (the so-called CIP 6/92). However, this scheme was open to both renewables and so-called ‘equivalent’ energy sources.

3 This boom soon represented a concern not only for policy makers, but also for companies and householders, as these costs were recovered via a surcharge on consumer bills (the so-called A3 charge).

4 According to ANEV, the Italian association of wind energy producers, the average time period to authorise a new wind installation is around five years, although law provisions provide for a maximum of six months (see Rinnovabili.it 2021).

5 The so-called “historical” cooperatives are exempt from surcharges and can offer cheaper electricity than privately owned companies. The cooperatives’ members are the owners and profits are passed on through cheaper prices to the final consumer.

6 The recitals of the IEMD and RED II explain what CECs and RECs are and their membership structure, governance requirements and purposes.

7 Eligible measures include the installation of new heating and cooling systems, building insulation, PV systems and electric charging stations.
